# Scoping review protocol of interventions for the mental health of women with and without HIV in Sub-Saharan Africa

**DOI:** 10.1136/bmjopen-2024-089266

**Published:** 2025-02-17

**Authors:** Lucas Banda, Mathildah Mpata Mokgatle, Olanrewaju Oladimeji

**Affiliations:** 1 Department of Social Sciences, Demography and Population Studies Unit, Walter Sisulu University, Mthatha, South Africa; 2 Department of Epidemiology and Biostatistics, Sefako Makgatho Health Sciences University, Pretoria, South Africa

**Keywords:** Psychological Stress, Depression & mood disorders, Anxiety disorders, Psychosocial Intervention, HIV & AIDS

## Abstract

**Abstract:**

**Introduction:**

Mental health issues among women in Sub-Saharan Africa (SSA), especially those living with HIV, pose a major public health challenge. Despite the established connections between HIV status and mental health outcomes, there is a noticeable absence of targeted interventions for this group within the literature. Many studies tend to focus on broad mental health concerns without addressing the specific needs of women with HIV, or they neglect to incorporate mental health elements into current HIV-related programmes. This scoping review aims to gather and analyse the existing research on interventions designed to improve the mental health of women in SSA, both with and without HIV. It will identify barriers preventing this population from accessing mental healthcare, highlight important gaps in the current literature and suggest directions for future research.

**Methods and analysis:**

To conduct this scoping review, the researcher will adhere to the methodological framework proposed by Arksey and O’Malley. The literature search will span several databases, including PubMed, MEDLINE, Web of Science and PsychInfo, to ensure a comprehensive collection of relevant studies. The selection process will involve two stages: two independent reviewers will initially screen titles for eligibility and a full-text review of the selected articles. A specially designed tool will be used for data extraction, focusing on minimising bias and accurately capturing study details. The final selection of studies will be analysed using a standardised tool to comprehensively assess all bibliographic information and study characteristics. The planned study dates for the review will be January to March 2025.

**Ethics and dissemination:**

No ethical approval is required as the review will draw on publicly available publications and materials. The study’s conclusions will be subject to peer review and published in a scientific journal, with the abstract shared at local and international conferences. Key findings will be disseminated to health ministries, community-based organisations focused on women’s mental health and HIV, and policymakers to inform policy decisions regarding mental health interventions for women in SSA.

**STRENGTH AND LIMITATIONS OF THIS STUDY**

- The review examines the intersection of mental health and HIV among women in Sub-Saharan Africa.
- Following the framework proposed by Arksey and O’Malley ensures a rigorous review process.
- A comprehensive search strategy across multiple electronic databases.
- The review is limited to English studies published from 2014 to 2023, which may exclude important non-English or older research.
- The scoping review does not critically appraise study quality, potentially introducing bias to findings.

## Introduction

 The significance of mental health interventions for women with and without HIV in Sub-Saharan Africa (SSA) cannot be overstated, given the unique sociocultural and economic challenges faced by this population. Women in this region disproportionately bear the burden of HIV/AIDS, with adverse mental health outcomes such as depression and anxiety exacerbated.^1^ For instance, a study revealed that almost one-third (31%) of people living with HIV in South Africa are experiencing mild-to-severe mental distress.^2^ Despite this pressing issue, there exists a notable gap in the literature regarding integrated and tailored interventions specifically designed for women dealing with both HIV and mental health challenges. Most existing studies tend to focus either on HIV/AIDS or mental health independently without considering the compounded effects of dealing with both.^3^,^7^ This study will focus on specific mental health conditions, particularly psychological distress, which includes depression and anxiety, as well as psychosocial stress.^8^

In addition, there is a significant gap in research regarding culturally sensitive mental health interventions aimed at addressing the specific challenges faced by women in SSA.^9 10^ This scoping review aims to fill that gap by synthesising existing literature on integrated interventions that specifically focus on the mental health needs of women, both with and without HIV, in this region. By examining studies that investigate the intersection of HIV and mental health, the review will underscore the necessity of developing tailored, culturally relevant interventions that take into account the unique sociocultural contexts and barriers encountered by women in SSA. Furthermore, the review will highlight effective implementation strategies and methodologies that have proven successful in other settings, providing a valuable framework for future research and intervention design that is relevant and adaptable to the specific needs of this population.

This scoping review seeks to clarify effective interventions for this vulnerable population by identifying and addressing specific gaps in current literature, including focusing on the barriers they face in accessing mental healthcare. Bridging this research gap is imperative for formulating comprehensive health policies, programmes and interventions to enhance these women’s quality of life and health.

## Methods and analysis

The planned study dates for the review will be January to March 2025. To assess psychological distress, studies that use several standardised diagnostic tools will be identified. These instruments are essential for capturing the variability in how depression and anxiety are assessed across studies and will help us understand their impact on intervention outcomes. The diagnostic tools include:

**Beck Depression Inventory:** A self-report measure assessing the severity of depression.

**Hamilton Depression Rating Scale:** A clinician-administered scale evaluating depression severity.

**Patient Health Questionnaire-9:** A self-administered tool screening for depression severity based on Diagnostic Statistical Manual (DSM) criteria.

**GeneralisedAnxiety Disorder 7-item scale:** A self-report questionnaire assessing anxiety severity.

**Kessler 10:** A 10-item scale measuring psychological distress.

**Clinical Diagnosis:** The clinical diagnosis could include the diagnosis made by the psychiatrist or clinical psychologist in population studies.

All primary peer-reviewed study designs, comprising qualitative and quantitative studies, will be considered in this review. On the other hand, inappropriate study designs or studies that do not fall within the specified designs of primary, secondary or peer-reviewed research (eg, case reports and anecdotal evidence) will be excluded to maintain methodological rigour. The scoping review will follow the methodological framework proposed by Arksey and O'Malley^11^ and the Joanna Briggs Institute scoping review methodology.^12^ The review will include the following stages. The review process consists of five steps, as shown in table 1.

**Table 1:** Scoping review protocol design, adapted from the framework proposed by Arksey and O’Malley^11^ with PRISMA-ScR

Arksey and O’Malley framework^11^	PRISMA-ScR^13^ protocol relevant checklist items
Identify the research questions	Objectives
Identifying relevant studies	Information sources, eligibility criteria and search strategy
Study selection	Selection of sources for evidence purpose
Charting data	Process of data charting, data items and critical appraisal
Collating, summarising and reporting findings	Summary of results

PRISMA-ScRPreferred Reporting Items for SystematicReviews and Meta-Analyses extension for Scoping Reviews

### Step 1: identifying the research question

A preliminary exploration of the literature was conducted to develop research questions. The primary research question focuses on identifying interventions for the mental health of women with and without HIV in SSA. Specifically, the research question is: what are the most effective interventions and barriers for improving the mental health of women, both with and without HIV, in the SSA context, considering the unique sociocultural and economic challenges they face?

The following are the specific objectives derived from the primary research question:

1. To analyse present mental health interventions and their effectiveness in enhancing psychological well-being among women with and without HIV.
2. To identify barriers that hinder access to mental health interventions for women in SSA, including sociocultural and economic factors.

### Step 2: identifying relevant studies

The study’s review will have peer-reviewed journal articles and grey literature information sources. Systematic search queries will be used for both groups using University online library sources. PubMed, MEDLINE, Web of Science and PsychInfo will be among the electronic databases used. In addition, the reference lists of studies that have been included will be scrutinised to uncover any relevant studies. Grey literature databases such as Open Grey and Grey Literature Report will be searched to unearth studies and reports pertinent to this review. The study’s search strategy will include the following keywords: scoping review, interventions, mental health, psychological distress, women, HIV, SSA and protocol. The study will limit the search to studies published in English from the last decade (2014–2023). Extensive and systematic grey literature will be presented to provide a balanced picture of the available evidence. Table 2 shows the source of grey literature and provides a commentary on how each source will be searched.

**Table 2:** Literature source, search engine and database

Grey literature database	GreyLitOpenGrey via DANS EASY
Peer reviewed articles	PubMed, Scopus, PsycInfo, Web of Science
Theses/dissertations	Proquest Dissertations and Theses GlobalWorldCat Dissertations and Theses
Internet search engines	Google and Google Scholar

### Search string

Table 3 provides a comprehensive outline of the search strategy. The lead researcher will determine pertinent search terms with the research team and an academic librarian. Keywords include scoping review, interventions, mental health, women, HIV, SSA and Protocol in the title, abstract and subject headings (ie, Medical Subject Headings, or MeSH, terms). The review articles retrieved will be screened for their titles, abstracts and index terms. Articles from each database will be imported into Zotero, a reference management software for record-keeping, article tracking and reference list creation, which will be incorporated into the final report.

**Table 3:** The examples of the search strategy that will be used to generate the articles to review for the research question

S/N	Database	Search term	Customisation
1	PubMed	(((trauma OR psychological distress OR mental-wellbeing OR depression OR anxiety OR mental illness OR psychosocial stress or mental health conditions) AND (pharmacological interventions* OR psychological interventions* OR psychosocial support* OR holistic approaches* OR alternative approaches* OR mental health strategies OR mental health programs)) AND (women OR females) AND ((AB (HIV OR Human Immunodeficiency Virus OR HIV/AIDS OR HIV-related disease OR people living with HIV OR HIV-positive)OR SU (HIV/AIDS)) AND (SSA OR Sub-Saharan Africa))))	English; (1 January 2014). −15 December, 2022
2	WOS	(((trauma OR psychological distress OR mental-wellbeing OR depression OR anxiety OR mental illness OR psychosocial stress or mental health conditions) AND (pharmacological interventions* OR psychological interventions* OR psychosocial support* OR holistic approaches* OR alternative approaches* OR mental health strategies OR mental health programs)) AND (women OR females) AND ((AB (HIV OR Human Immunodeficiency Virus OR HIV/AIDS OR HIV-related disease OR people living with HIV OR HIV-positive)OR SU (HIV/AIDS)) AND (SSA* OR Sub-Saharan Africa))))	English; (1 January 2014). −15 December, 2022
3	SCOPUS	(TITLE-ABS-KEY (trauma OR psychological distress OR mental-wellbeing OR depression OR anxiety OR mental illness OR psychosocial stress or mental health conditions) AND TITLE-ABS-KEY (pharmacological interventions* OR psychological interventions* OR psychosocial support* OR holistic approaches* OR alternative approaches* OR mental health strategies OR mental health programs) AND TITLE-ABS-KEY (women OR females) AND TITLE-ABS-KEY (AB (HIV OR Human Immunodeficiency Virus OR HIV/AIDS OR HIV-related disease OR people living with HIV OR HIV-positive)OR SU (HIV/AIDS) AND TITLE-ABS-KEY (SSA OR Sub-Saharan Africa))	English; (1 January 2014). −15 December, 2022
4	PsychoInfo	(AB (trauma OR psychological distress OR mental well-being OR depression OR anxiety OR mental illness OR psychosocial stress OR mental health conditions)OR TI (trauma OR psychological distress OR mental well-being OR depression OR anxiety OR mental illness OR psychosocial stress OR mental health conditions))AND (AB (pharmacological interventions* OR psychological interventions* OR psychosocial support* OR holistic approaches* OR alternative approaches* OR mental health strategies OR mental health programs))AND ((AB (HIV OR Human Immunodeficiency Virus OR HIV/AIDS OR HIV-related disease OR people living with HIV OR HIV-positive)OR SU (HIV/AIDS))AND (AB (women) OR SU (women)))AND (TI (SSA) OR AB (Sub-Saharan Africa))	English; (1 January 2013). −15 December, 2022

### Synthesis of eligibility criteria

Table 4 will feature all peer-reviewed, published studies that outline the interventions for the mental health of women with and without HIV in SSA, encompassing the following inclusion criteria: (1) published in English, (2) within the timeframe of 2014–2023. The period from 2014 to 2023 was selected for our scoping review because it corresponds with the first Ten-Year Implementation Plan of Agenda 2063. This timeframe reflects modern interventions and strategies in mental health that are pertinent to current policies in SSA. During these years, we have witnessed significant progress in mental health research and the integration of mental health services into HIV care. This allows us to explore emerging trends and their effects on women living with and without HIV. Furthermore, there has been a marked increase in studies focused on mental health issues in SSA during this period, providing a more extensive body of literature for our review. (3) Conducted in any setting: the search strategy aims to encompass studies from a range of settings, with a primary emphasis on literature that pertains to SSA. By adopting this broad approach, we can include a variety of interventions that could be relevant or adaptable to the context of SSA, especially those studies conducted in similar sociocultural environments (4) consisting of randomised controlled trials, intervention studies (including quasi-experimental designs), qualitative and quantitative studies, and (5) examining women with and without HIV who may have experienced psychological distress or adverse mental health conditions. Any studies that fail to meet the criteria will be excluded from identifying Interventions for the mental health of women with and without HIV in SSA.

**Table 4:** Inclusion and exclusion criteria for the scoping review

S/N	Category	Inclusion criteria	Exclusion criteria
1	Language	English	
2	Publication date range	January 2014 to December 2023	Before 2014
3	Participant age	Women of reproductive age (15–49 y)	
4	Population category	Women with and without HIV	Men; women not within the specified age range
5	Studies	This scoping review will focus on interventions designed to enhance mental well-being for women in SSA, both with and without HIV.	Studies are not focused on mental health interventions but outside the specified population category. This includes studies that address health issues or interventions outside the realm of mental health, such as those primarily focused on physical health conditions, non-mental health-related social services or general wellness programmes that do not specifically target mental health outcomes.
6	Study design	All designs of primary, secondary and peer-reviewed studies, including randomised control trials, intervention studies (including quasi-experimental designs), qualitative and quantitative studies, systematic reviews scoping reviews, scoping reviews and meta analyses.	Inappropriate study designs: studies that do not fall within the specified designs of primary, secondary or peer-reviewed research (eg, case reports, anecdotal evidence) will be excluded to maintain methodological rigour.

Publication date: January 2014 to December 2023. Language: English. Interventions for the Mental Health of Women with and without HIV in Sub-Saharan Africa

### Setting

This review will encompass studies that outline the Interventions for the mental health of women with and without HIV in SSA.

#### Step 3: study selection

**Figure 1:**
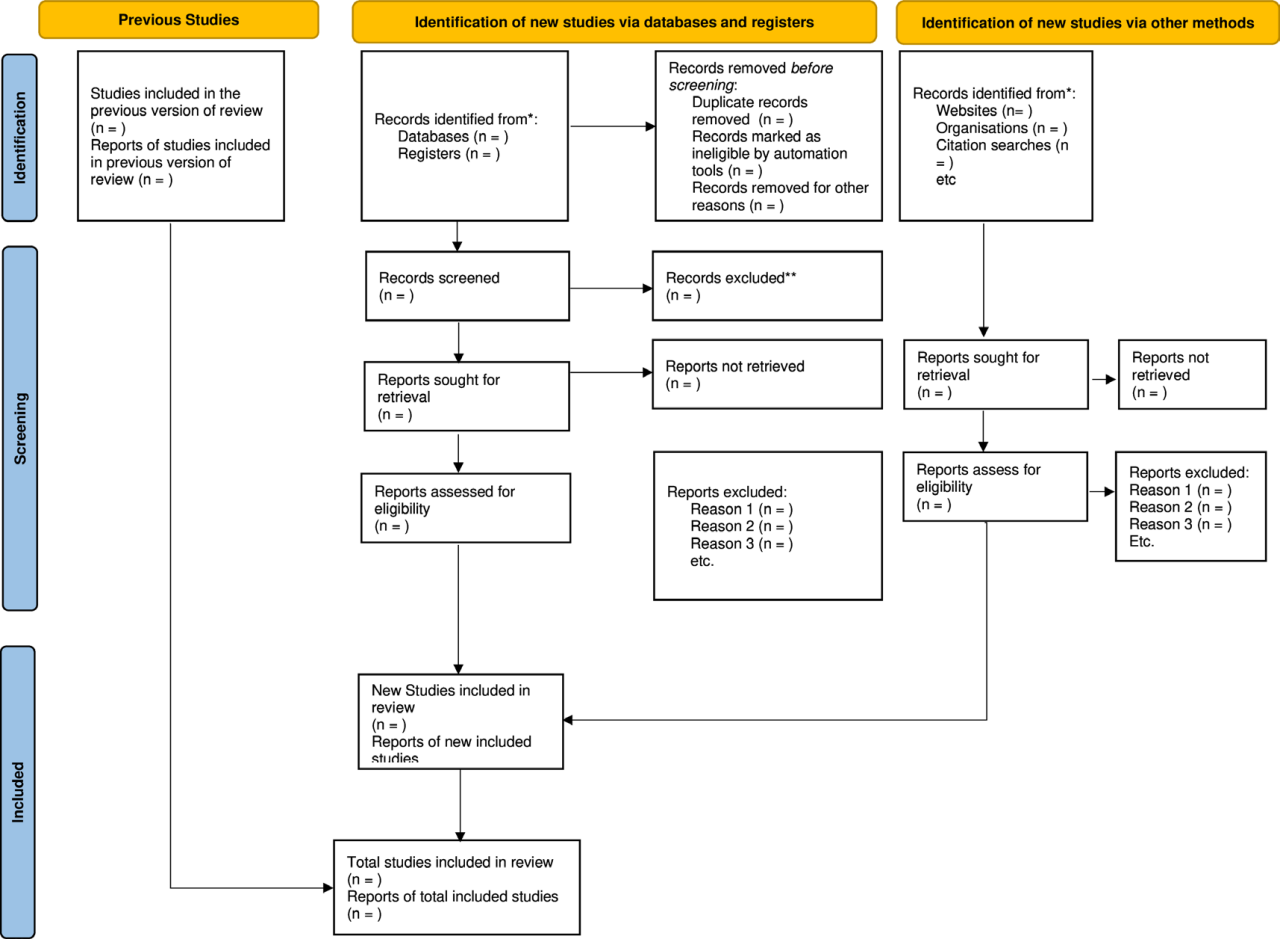
Preferred Reporting Items for Systematic Reviews and Meta-Analyses flowchart.^14^

Two reviewers will independently evaluate the identified studies’ titles and abstracts in this scoping review. The inclusion criteria are studies examining the Interventions for the mental health of women with and without HIV in SSA, published in English and with full-text availability. In the event of discrepancies between the reviewers, they will convene and reach a mutual agreement via discussion. The article screening process for this review will comprise two stages. First, two team members will independently assess the titles and abstracts of all retrieved citations for eligibility against the established inclusion criteria (table 3). We will scan their reference lists to see if relevant meta-analyses or reviews have been identified. Two team members will independently screen a sample of retrieved articles to ensure the eligibility criteria are uniformly applied. Articles deemed relevant by one or both reviewers will be included in the full-text review. The inter-rater reliability will be evaluated using the percentage agreement. The inclusion and exclusion criteria will be clarified if the percentage agreement is less than 80%. The second stage will involve removing duplicate records from different databases. Then, a team member will screen the titles and abstracts of the remaining articles to exclude those that do not meet the predetermined eligibility criteria. The titles and abstracts that satisfy the inclusion criteria will be reviewed thoroughly. Any disagreements regarding eligibility will be resolved through discussion among reviewers or with the intervention of a third party if necessary. The study selection process will be documented using the Preferred Reporting Items for Systematic Reviews and Meta-Analyses flowchart (figure 1).

Although manual screening is certainly an option, we understand that it can be both time-consuming and prone to errors, particularly with large datasets. To improve our screening process, we will adopt several strategies aimed at enhancing both efficiency and accuracy. Although we recognise the limitations of a manual approach, these measures are designed to boost the reliability of our screening and ensure that relevant studies are correctly identified for inclusion in our scoping review.

#### Step 4: charting the data

Using a standardised data extraction form, two reviewers will independently extract data from the included studies. This form will capture critical information such as the author, year of publication, study design, study setting, sample size and the specific types of interventions. In addition, the form will document the characteristics of the study population, the study method and the main findings of each study. Based on the initial scoping phase, a data extraction framework will be developed to guide eligible full text retrieved in the literature. This framework will be updated regularly as we conduct the full-text review. This will allow us to include any new categories or important information that comes up from the studies we're examining.

Each article selected for review will be assessed for relevance based on the publication date falling between January 2014 and December 2023. The study population of interest will consist of women aged 15–49 years, encompassing those with and without HIV. The focus will be on interventions targeting mental health outcomes in this demographic. Various study designs will be considered, including randomised control trials, intervention studies and qualitative or quantitative research, while excluding meta-analyses and systematic reviews. To ensure consistency, two team members will pilot-test a sample of the included studies and modify categories if new ones emerge. Any disagreements will be resolved through team consultations and authors of eligible abstracts with missing information will be contacted. The responsibility for charting the data from each included study will be placed on the team members piloting the framework. Any disagreements in data extracted by the two team members will be discussed until a consensus is reached or by arbitration of a third reviewer if required.

#### Step 5: Collating, summarising and reporting of findings

The scoping review will focus on mapping out the range of interventions aimed at improving the mental health of women with and without HIV in SSA. This review aims to consolidate and summarise the existing knowledge concerning these interventions by using a narrative synthesis approach, highlighting the strategies that have effectively addressed this population’s mental health needs. The review will explore the various interventions identified from the included studies, contributing to the knowledge base by summarising, interpreting and reporting these interventions using a standardised approach. Furthermore, it will identify gaps in the current literature and suggest areas for future research.

Rather than focusing on the quality of individual studies, the review will provide a comprehensive overview of the evidence, compiling results and presenting a structured summary. The findings from this scoping review will be instrumental in developing targeted strategies to understand better and enhance the interventions available for the mental health of women with and without HIV in this region, acknowledging the high prevalence and impact of HIV.

## Discussion

### Anticipated outputs and implications

The expected results of this scoping review will offer a thorough overview of mental health interventions for women living with and without HIV in SSA. By analysing the current literature, we aim to shed light on the various types of available interventions, their effectiveness and the accessibility challenges this population faces.

### Identifying gaps in literature

One of the primary objectives of this review is to identify gaps in the current literature on mental health interventions for women in SSA. Through the systematic examination of the available studies, the study will highlight areas where research is lacking, especially concerning culturally sensitive interventions and those specifically tailored to the unique socioeconomic challenges faced by women in this region.

### Direction for future research

The findings from this scoping review will serve as a foundation for future research initiatives. By highlighting gaps in knowledge, the study will provide researchers, policymakers and practitioners with direction on where further investigation is needed. This may include exploring innovative intervention strategies, assessing long-term outcomes of existing programmes or examining the role of community support systems in enhancing mental health outcomes.

## Patient and public involvement

None.

## Ethics and dissemination

No ethical approval is required as the review will draw on publicly available publications and materials. The study’s conclusions will be subject to peer review and published in a scientific journal, with the abstract shared at local and international conferences. The review’s findings will be disseminated to health ministries, community-based organisations focused on women’s mental health and HIV, and policymakers to inform policy decisions regarding mental health interventions for women in SSA. The insights gathered from this review are aimed at serving as an essential resource for policymakers, researchers and healthcare practitioners. They can use this information to develop well-informed, targeted interventions to strengthen women’s mental health in SSA, thereby addressing a vital aspect of their overall well-being.
