# Supplementary material for: Scoping review protocol of interventions for the mental health of women with and without HIV in Sub-Saharan Africa
Source: BMJ Open. 2025 Feb 17;15(2):e089266. doi: 10.1136/bmjopen-2024-089266 (PMC11836823; doi:10.1136/bmjopen-2024-089266)
Supplement: Supplementary file 1 [file bmjopen-2024-089266.reviewer_comments.pdf]

PEER REVIEW HISTORY

BMJ Open publishes all reviews undertaken for accepted manuscripts. Reviewers are asked to complete a checklist review form and are provided with free text boxes to elaborate on their assessment. These free text comments are reproduced below.

ARTICLE DETAILS

Title (Provisional)

Scoping Review Protocol of Interventions for the Mental Health of Women with and without HIV in Sub-Saharan Africa

Authors

Banda, Lucas; Mokgatle, Mathildah Mpata; Oladimeji, O

VERSION 1 - REVIEW

**Reviewer**	**1**
**Name**	**Adjorlolo, Samuel**
**Affiliation**	**University of Ghana**
**Date**	**12-Sep-2024**
**COI**	**None declared**

Abstract.

1. The introduction does not provide a clear, focused rationale for the scoping review. It mentions the need for a "comprehensive understanding of effective interventions" without specifying the gaps in the current literature.
2. Was the protocol prospectively registered in any of the accredited registries? If so, please provide the protocol registration number.

Strengths and limitations of the study

3. Page 2, line 20, incomplete spelling of ‘strength’
4. Page 2 line 22: The first strength is neither substantial nor compelling. The authors should clarify what distinct insights this review will provide that previous reviews have not.
5. Page 2 Line 45: The second limitation about not conducting a critical appraisal of study quality is a fair point, but the authors do not explain how this might impact the reliability and completeness of the synthesized findings.

Introduction:

6. The introduction provides a lot of background information on the significance of mental health challenges for women with and without HIV in sub-Saharan Africa, but it does not clearly articulate the specific gaps in the literature that this scoping review aims to address.
7. On page 3, lines 23 – 24, the authors mentioned “Most existing studies tend to focus either on HIV/AIDS or mental health independently …” without providing any evidence to substantiate the claim.

8. On page 3 line 22 and 28, The authors mention the lack of "integrated and tailored interventions" and "culturally sensitive interventions," but it does not clearly explain how the proposed scoping review will address these gaps.
9. Page 3 line 31/32, The authors bring up the potential use of population data and advanced technologies, such as machine learning, for mental health interventions, but it does not clearly explain how this relates to the objectives of the scoping review.

Methods and Analysis:

10. Different citation style: Line 59, page 3 “Arksey and O'Malley (2005)”
11. Step 1 – Identifying the research question
12. Page 4 line 36/37: Although step 1 outlines what the authors should present, the research question remains unclear. Instead, they have provided the study's overall objective.

Specific objectives

13. General comment: The objectives are quite broad and cover a wide range of aspects related to mental health interventions for women (with and without HIV) in sub-Saharan Africa. This broad scope may make it challenging to conduct a focused and in-depth review within the constraints of a scoping review.

Specific comments:

14. The objective to "explore how community and social support systems influence the mental health of women" is quite open-ended and may be difficult to comprehensively address within the scoping review framework.
15. The objective to "review existing policies and frameworks" goes beyond the typical scope of a scoping review, which is more focused on identifying and mapping the existing literature.
16. Page 5, lines 23 – 26 appear as part of objective 7 but it reads as the main research question. I suggest it is stated distinctly.

Step 2: Identifying relevant studies.

17. General comment: It is clear from this study that the authors did not follow any framework (PICO, PCC) to develop/generate the search strategy and eligibility criteria. On page 6, it is unclear what framework the authors used to identify the keywords for the search (e.g. PICO, PCC, etc.) as this was not stated or implied

Specific comments:

18. Page 5 line 44: Why include ‘scoping review’ and ‘protocol’ (lines 44 and 45, page 5) as key words for the search strategy when your eligibility criteria exclude all reviews?
19. Page 5, Table 2: Articles indexed in these electronic databases (PubMed, Scopus, PsycInfo, Web of Science) are not grey but peer-reviewed. Delete the “Grey and published articles” and make it Peer reviewed articles.
20. Page 6, Table 3: The search may miss relevant articles that do not use the exact terms specified, such as "psychological distress" or "mental well-being". More synonyms or related terms could be included to broaden the search.
21. On Table 3, pages 6 and 7, the application of truncation (*) and quotation marks (“ ”) is questionable. For instance, mental health*, interventions*, “women”. Truncation is commonly used when you want to retrieve variations of a word root. For example, **Child*** would retrieve results for child, children, childhood, etc. Also, quotation marks are applied to more than one word (phrase) to retrieve results that match the exact **phrase** in the exact order and combination as they appear within the quotes. For example, searching for **“mental**

health intervention" will return results where this exact phrase is used, instead of results where the words "mental," "health," and "intervention" appear separately.

22. Table 3, page 6, there is no evidence of mesh terms in PubMed (search strategy) which is likely to limit the search.
23. Page 6, Table 3: The search does not appear to include terms related to intervention types or strategies. More specific intervention-related terms could help target the search to the intended scope.
24. The search does not seem to include terms related to the mental health outcomes of interest, such as depression, anxiety, trauma, etc. Adding these types of terms could help focus the search on the specific mental health issues being examined
25. Broaden the HIV-related terms
26. More synonyms or related terms on mental health, psychological distress could be included to broaden the search.
27. Page 7 table 4: The eligibility criteria mention studies from 2014 to 2023. The authors could provide the rationale for considering this timeline so that readers can better appreciate it (PRISMA-ScR Checklist, item 6).
28. In Line 24, page 7, point (iii), the authors mentioned the search will be conducted in any setting which is contrary to the SSA context mentioned in line 20 of the same page (and throughout the text). This needs to be clarified.
29. Line 28, page 7, point (v) "… who may have experienced psychological distress or adverse mental health outcomes …" This statement appears out of the blue and does not appear in other related fields e.g. Table 4
30. Table 4, exclusion criteria under point 5 is unclear.

Step 3 -Study selection

31. It appears the authors intend to screen identified articles manually without any screening tool (like Rayyan or Covidence). Although this is possible it may be time-consuming and error-prone, especially with large datasets

Step 4 – Charting the data

32. The data extraction framework was developed based on an initial scoping phase, but it is unclear if this framework will be iteratively updated as the full-text review progresses.
33. There was no heading on discussion as required by the PRISMA-ScR guidelines for reporting (criterion 19 and 20). The study does not provide any information about the anticipated outputs or implications of the scoping review. There is no discussions of how the findings will be used to identify gaps in the literature or provide directions for future research.

**Reviewer**	**2**
**Name**	**van de Vijver, David**
**Affiliation**	**Erasmus MC, Viroscience**
**Date**	**23-Sep-2024**
**COI**	**Nothing to declare**

Lucas Banda and colleagues describe a scoping review protocol of interventions for mental health of women with and without HIV in sub-Saharan Africa. This is a very important topic that deserves attention. The protocol that is described is very thorough. I have a few suggestions.

1. The authors write that they will focus on mental health which is rather broad. I would suggest focusing on only one (or a few) mental health condition as the review may become too heterogeneous.

2. The authors should include the method by which a mental health diagnosis is made as these can differ and can have an impact on the outcome. For instance, for depression I can already name a few such as the Beck Depression Inventory, Hamilton depression rating scale, DSM V, and then studies may also base depression on a diagnosis made by a psychiatrist. Please also include the severity of the mental condition (if known).

VERSION 1 - AUTHOR RESPONSE

Reviewer Comments	Response (How Addressed in the Manuscript)
**Reviewer 1 - Technical Contribution:**<br><br>Abstract.<br>1. The introduction does not provide a clear, focused rationale for the scoping review. It mentions the need for a "comprehensive understanding of effective interventions" without specifying the gaps in the current literature.	1. The abstract has been revised and it addresses gaps in literature and to justify the scoping review study.
2. Was the protocol prospectively registered in any of the accredited registries? If so, please provide the protocol registration number.	2. Thank you for the reviewer 1's feedback regarding the registration of the scoping review protocol. I would like to clarify that, to our knowledge, registering a protocol for scoping reviews is not mandatory, unlike systematic reviews. However, it is increasingly recognized as a best practice to enhance transparency and reduce bias in research.<br>Currently, scoping reviews can be registered with platforms such as the Open Science Framework (OSF) or Figshare, but they are not typically registered in databases like PROSPERO, which is primarily for systematic reviews. As of now, I have

**Strengths and limitations of the study**<br>3. Page 2, line 20, incomplete spelling of 'strength'<br><br>4. Page 2 line 22: The first strength is neither substantial nor compelling. The authors should clarify what distinct insights this review will provide that previous reviews have not.<br>5. Page 2 Line 45: The second limitation about not conducting a critical appraisal of study quality is a fair point, but the authors do not explain how this might impact the reliability and completeness of the synthesized findings.<br><br>**Introduction:**<br>6. The introduction provides a lot of background information on the significance of mental health challenges for women with and without HIV in sub-Saharan Africa, but it does not clearly articulate the specific gaps in the literature that this scoping review aims to address.<br>7. On page 3, lines 23 – 24, the authors mentioned "Most existing studies tend to focus either on HIV/AIDS or mental health independently ..." without providing any evidence to substantiate the claim.<br>8. On page 3 line 22 and 28, The authors mention the lack of "integrated and tailored interventions" and "culturally sensitive interventions," but it does not clearly explain how the proposed scoping review will address these gaps.<br>9. Page 3 line 31/32, The authors bring up the potential use of population data and advanced technologies, such as machine learning, for mental health interventions, but it	not registered the protocol in any accredited registries.<br>If there are specific registries you recommend for scoping reviews, I would be open to considering them for future projects.<br><br>3. The spelling of STRENGTH in the subheading after abstract has been corrected.<br><br>4. This has been addressed in the first strength of the study.<br><br>5. The second limitation of the study, which is the last point in the strength and limitations section has also been addressed to indicate that lack of appraisal may lead to bias in the findings.<br><br>6. The introduction has been revised and it addresses gaps in literature and to justify the scoping review study.<br><br>7. Evidence to substantiate our claim has been added.<br><br>8. This has been addressed in the introduction section on the section on the cultural relevance of the interventions.<br><br>9. This has ben addressed and justified: While the primary objectives focus on mapping existing interventions, accessibility, effectiveness, barriers, community support, and policy frameworks,

does not clearly explain how this relates to the objectives of the scoping review.	incorporating an examination of machine learning will provide valuable insights into how these technologies can be utilized to analyze data patterns, predict mental health outcomes, and tailor interventions to meet the specific needs of this population. This approach aligns with our objective to identify gaps in current literature and suggest areas for future research, ultimately aiming to improve the design and implementation of culturally sensitive and effective mental health strategies.
**Methods and Analysis:**<br>10. Different citation style: Line 59, page 3 “Arksey and O'Malley (2005)”	10. Addressed by removing 2005 and maintaining the citation style that has been throughout the document.
**11. Step 1 – Identifying the research question**<br>12. Page 4 line 36/37: Although step 1 outlines what the authors should present, the research question remains unclear. Instead, they have provided the study's overall objective.	12. The research question has been provided.
Specific objectives<br>13. General comment: The objectives are quite broad and cover a wide range of aspects related to mental health interventions for women (with and without HIV) in sub-Saharan Africa. This broad scope may make it challenging to conduct a focused and in-depth review within the constraints of a scoping review.	13 In response to the reviewer’s comment regarding the broadness of our objectives, we have streamlined our objectives and have focused on review of interventions and barriers to mental healthcare or access to these intervention. This adjustment ensures a more focused approach while still addressing critical aspects of mental health interventions for women living with and without HIV in Sub-Saharan Africa.
	14. We have removed this objective as the thorough analysis of the

	paper revealed that it was outside our scope of what the review wants to do.
Specific comments:<br>14. The objective to "explore how community and social support systems influence the mental health of women" is quite open-ended and may be difficult to comprehensively address within the scoping review framework.	15. It is acknowledged that this objective could be outside of the scope of this scoping review and it has been removed.
15. The objective to "review existing policies and frameworks" goes beyond the typical scope of a scoping review, which is more focused on identifying and mapping the existing literature.	16. This has been addressed adequately as the main research question that was misplaced.
16. Page 5, lines 23 – 26 appear as part of objective 7 but it reads as the main research question. I suggest it is stated distinctly.	
**Step 2: Identifying relevant studies.**<br>17. General comment: It is clear from this study that the authors did not follow any framework (PICO, PCC) to develop/generate the search strategy and eligibility criteria. On page 6, it is unclear what framework the authors used to identify the keywords for the search (e.g. PICO, PCC, etc.) as this was not stated or implied<br>Specific comments:	17. In developing the search strategy for this scoping review, we recognize the importance of using a structured framework to guide our approach. While we initially did not explicitly state the use of PICO or PCC, we will clarify that our search strategy is informed by the PCC framework (Population, Concept, Context) rather than PICO, as it aligns more closely with the objectives of a scoping review.<br>**Population:** The target population includes women living with and without HIV in Sub-Saharan Africa.<br>**Concept:** The primary concept focuses on mental health interventions aimed at improving mental health outcomes for this demographic.<br>**Context:** The context encompasses various socio-cultural and economic factors relevant to Sub-Saharan Africa.

18. Page 5 line 44: Why include ‘scoping review’ and ‘protocol’ (lines 44 and 45, page 5) as key words for the search strategy when your eligibility criteria exclude all reviews?	18. Regarding the inclusion of keywords such as "scoping review" and "protocol," we acknowledge that this was an oversight since our eligibility criteria exclude systematic reviews and meta-analyses. We have revised our keywords selection to ensure that it accurately reflects our focus on primary research studies related to mental health interventions.
19. Page 5, Table 2: Articles indexed in these electronic databases (PubMed, Scopus, PsycInfo, Web of Science) are not grey but peer-reviewed. Delete the “Grey and published articles” and make it Peer reviewed articles.	19. Addressed in Table 2<br><br>emotional well-being," "mental illness," "psychosocial stress," and "mental health conditions
20. Page 6, Table 3: The search may miss relevant articles that do not use the exact terms specified, such as "psychological distress" or "mental well-being". More synonyms or related terms could be included to broaden the search.	20. More related terms have been used to broaden the search results in Table 3.
21. On Table 3, pages 6 and 7, the application of truncation (*) and quotation marks (“ ”) is questionable. For instance, mental health*, interventions*, “women”. Truncation is commonly used when you want to retrieve variations of a word root. For example, Child* would retrieve results for child, children, childhood, etc. Also, quotation marks are applied to more than one word (phrase) to retrieve results that match the exact phrase in the exact order and combination as they appear within the quotes. For example, searching for “mental health intervention” will return results where this exact phrase is	21. We want to appreciate the reviewer for taking time to explain the appropriate use of truncation and quotation marks. The revision has been made in Table 3.
	22. In response to the reviewer's comment about the lack of MeSH

used, instead of results where the words "mental," "health," and "intervention" appear separately.<br><br>22. Table 3, page 6, there is no evidence of mesh terms in PubMed (search strategy) which is likely to limit the search.	terms in our search strategy, we acknowledge the importance of incorporating these controlled vocabulary terms to broaden our search and improve the retrieval of relevant literature. We have utilized the following MeSH terms relevant to our study:<br>• **Mental Health**<br>• **Depression**<br>• **Anxiety**<br>• **HIV Infections**<br>• **Psychological Distress**
23. Page 6, Table 3: The search does not appear to include terms related to intervention types or strategies. More specific intervention-related terms could help target the search to the intended scope.	23. More search terms for intervention, including truncation, have been used to extrapolate different types of interventions. For example, using truncation for Psycho-social Support, may provide literature on peer support, community-based interventions, and family therapy. Additionally, using truncation for intervention such as Holistic and Alternative Approaches could give us literature on mindfulness, yoga, art therapy or music therapy.<br><br>24. This has already been addressed in the search strategy.
24. The search does not seem to include terms related to the mental health outcomes of interest, such as depression, anxiety, trauma, etc. Adding these types of terms could help focus the search on the specific mental health issues being examined<br><br>25. Broaden the HIV-related terms	25. HIV-related terms have been used.

26. More synonyms or related terms on mental health, psychological distress could be included to broaden the search.<br>27. Page 7 table 4: The eligibility criteria mention studies from 2014 to 2023. The authors could provide the rationale for considering this timeline so that readers can better appreciate it (PRISMA-ScR Checklist, item 6).<br><br>28. In Line 24, page 7, point (iii), the authors mentioned the search will be conducted in any setting which is contrary to the SSA context mentioned in line 20 of the same page (and throughout the text). This needs to be clarified.<br><br>29. Line 28, page 7, point (v) "… who may have experienced psychological distress or adverse mental health outcomes …" This statement appears out of the blue and does not appear in other related fields e.g. Table 4<br><br>30. Table 4, exclusion criteria under point 5 is unclear.<br><br>**Step 3 -Study selection**<br><br>31. It appears the authors intend to screen identified articles manually without any screening tool (like Rayyan or Covidence). Although this is possible it may be time-consuming and error-prone, especially with large datasets	26. These have been addressed already in the search strategy.<br>27. The rationale has been provided aligning it with PRISMA-Checklist, item 6 that requires the rationale be given for the selection of time period for studies.<br><br>28. The point number iii mentioned that the study will be conducted in any setting has been clarified.<br><br>29. These conditions have now been added in search strategy and Table 4.<br><br>30. The exclusion criteria has been clarified.<br><br>31. While manual screening is indeed possible, we recognize that it may be time-consuming and prone to errors, especially with large datasets.<br>To enhance our screening process, we will implement several strategies to improve efficiency and accuracy. While we acknowledge the limitations of a manual approach, these measures aim to enhance the reliability of our screening process and ensure that relevant studies are accurately identified for inclusion in our scoping review.

**Step 4 – Charting the data**<br>32. The data extraction framework was developed based on an initial scoping phase, but it is unclear if this framework will be iteratively updated as the full-text review progresses.<br><br>33. There was no heading on discussion as required by the PRISMA-ScR guidelines for reporting (criterion 19 and 20). The study does not provide any information about the anticipated outputs or implications of the scoping review. There is no discussions of how the findings will be used to identify gaps in the literature or provide directions for future research.	32. This had been updated thus in the text on charting the data:<br>"This framework will be updated regularly as we conduct the full-text review. This will allow us to include any new categories or important information that comes up from the studies we're examining."<br><br>33. A discussion that includes anticipated outputs, gaps in literature, and direction for future research has been added.
**Reviewer 2**<br>1. The authors write that they will focus on mental health which is rather broad. I would suggest focusing on only one (or a few) mental health	1. We appreciate the reviewer's suggestion regarding the focus of our review. In our study, we will specifically target psychological distress, which encompasses depression, anxiety, and psychosocial stress. These conditions are prevalent and significant mental health issues affecting women living with and without HIV in Sub-Saharan Africa. While our search strategy includes a broader range of terms such as trauma, mental well-being, mental illness, mental health conditions and psychological distress, we will focus our analysis on the specified conditions of psychological distress, depression, anxiety, and psychosocial stress. This focused approach allows us to provide a more in-depth exploration of interventions specifically addressing these critical mental health issues.

2. The authors should include the method by which a mental health diagnosis is made as these can differ and can have an impact on the outcome. For instance, for depression I can already name a few such as the Beck Depression Inventory, Hamilton depression rating scale, DSM V, and then studies may also base depression on a diagnosis made by a psychiatrist. Please also include the severity of the mental condition (if known).	2. We appreciate your insightful feedback regarding the need to include information about the methods used for diagnosing mental health conditions and their severity. In our study, we will consider various standardized assessment tools commonly used to diagnose psychological distress, particularly depression and anxiety. These tools include:<br>**Beck Depression Inventory (BDI):** A widely used self-report measure that assesses the severity of depression.<br>**Hamilton Depression Rating Scale (HDRS):** A clinician-administered scale that evaluates depression severity based on patient interviews.<br>**Diagnostic and Statistical Manual of Mental Disorders (DSM-5):** A classification system that provides criteria for diagnosing mental health disorders, including depression.<br>**Patient Health Questionnaire-9 (PHQ-9):** A self-administered tool that screens for depression severity based on DSM criteria.<br>**Kessler 10 (K10):** A 10-item scale designed to measure psychological distress, specifically assessing symptoms of anxiety and depression over the past 30 days. It provides a total score indicating levels of distress, making it a valuable tool for screening in both clinical and general populations.<br>By incorporating these diagnostic methods, we aim to capture the variability in how depression and anxiety are assessed across studies.

VERSION 2 - REVIEW

**Reviewer**	**1**
**Name**	**Adjorlolo, Samuel**
**Affiliation**	**University of Ghana**
**Date**	**13-Jan-2025**
**COI**	

My comments have been addressed.
